# Functional interactions of the cystine/glutamate antiporter, CD44v and MUC1-C oncoprotein in triple-negative breast cancer cells

**DOI:** 10.18632/oncotarget.7598

**Published:** 2016-02-22

**Authors:** Masanori Hasegawa, Hidekazu Takahashi, Hasan Rajabi, Maroof Alam, Yozo Suzuki, Li Yin, Ashujit Tagde, Takahiro Maeda, Masayuki Hiraki, Vikas P. Sukhatme, Donald Kufe

**Affiliations:** ^1^ Dana-Farber Cancer Institute, Harvard Medical School, Boston, MA, USA; ^2^ Beth Israel Deaconess Medical Center, Harvard Medical School, Boston, MA, USA; ^3^ Department of Gastrointestinal Surgery, Graduate School of Medicine, Osaka University, Suita City, Osaka, Japan; ^4^ Department of Gastroenterological Surgery, Osaka Police Hospital, Osaka City, Osaka, Japan

**Keywords:** xCT, MUC1-C, CD44v, epigenetics, ferroptosis

## Abstract

The xCT light chain of the cystine/glutamate transporter (system X_C_^−^) is of importance for the survival of triple-negative breast cancer (TNBC) cells. The MUC1-C transmembrane oncoprotein is aberrantly overexpressed in TNBC and, like xCT, has been linked to maintaining glutathione (GSH) levels and redox balance. However, there is no known interaction between MUC1-C and xCT. Here we show that silencing MUC1-C is associated with decreases in xCT expression in TNBC cells. The results demonstrate that MUC1-C forms a complex with xCT and the CD44 variant (CD44v), which interacts with xCT and thereby controls GSH levels. MUC1-C binds directly with CD44v and in turn promotes stability of xCT in the cell membrane. The interaction between MUC1-C and xCT is further supported by the demonstration that targeting xCT with silencing or the inhibitor sulfasalazine suppresses *MUC1* gene transcription by increasing histone and DNA methylation on the *MUC1* promoter. In terms of the functional significance of the MUC1-C/xCT interaction, we show that MUC1-C protects against treatment with erastin, an inhibitor of X_C_^−^ and inducer of ferroptosis, a form of non-apoptotic cell death. These findings indicate that targeting this novel MUC1-C/xCT pathway could represent a potential therapeutic approach for promoting TNBC cell death.

## INTRODUCTION

The system X_C_^−^ cystine/glutamate antiporter is a member of the family of heterodimeric amino acid transporters. System X_C_^−^ is composed of a light chain, designated xCT, and the 4F2 heavy chain that traffics the heterodimer to the cell membrane [1]. xCT functions as a major transporter for the uptake of cystine in exchange for glutamate. xCT-mediated uptake of cystine and its intracellular reduction to cysteine, constitute a redox couple that maintains balance between extracellular cystine and cysteine [1]. Additionally, intracellular cysteine is the rate-limiting precursor for the synthesis of glutathione (GSH), a tripeptide consisting of glutamate, cysteine and glycine, that plays a critical role in the cellular defense against oxidative stress [2]. As a result, cells from xCT-deficient mice exhibit decreases in cysteine and GSH levels [3]. xCT is expressed by diverse cancer cells and contributes to their growth, survival and resistance to anti-cancer agents, at least in part, by maintaining GSH levels and thereby redox balance [4-6]. The CD44 variant (CD44v), a marker of cancer stem-like cells (CSCs), stabilizes xCT at the cell membrane and thus enhances cystine uptake for GSH synthesis in gastrointestinal cancer cells [7]. Moreover, ablation of CD44 suppresses xCT function, induces oxidative stress and inhibits gastric tumor growth in transgenic mice [7]. Other work has shown that xCT is expressed in triple-negative breast cancers (TNBC) and contributes to glutamine dependency [8]. In this way, conversion of glutamine to glutamate is needed for xCT-mediated uptake of cystine. Inhibition of xCT in TNBC cells with the clinically approved anti-inflammatory agent sulfasalazine (SASP) decreases cystine uptake, GSH production and tumor growth [8]. These and other findings that chemotherapy induces xCT expression in TNBC cells [9] have supported the notion that xCT represents a potential target for cancer cell-specific GSH depletion and, in turn, sensitization to treatment with anti-cancer agents that disrupt redox balance [5, 10, 11].

The mucin 1 (MUC1) transmembrane glycoprotein is aberrantly overexpressed in most breast cancers, including about 90% of TNBCs [12, 13]. MUC1 consists of two subunits that form a heterodimeric complex at the cell membrane [12]. The MUC1 N-terminal subunit (MUC1-N) contains glycosylated tandem repeats that are characteristic of the mucin family. The oncogenic MUC1 C-terminal subunit (MUC1-C) consists of a 58 amino acid (aa) extracellular domain, a 28 aa transmembrane region and a 72 aa cytoplasmic tail [12]. Overexpression of the MUC1-N/MUC1-C complex has been associated with amplification of the *MUC1* gene at chromosome 1q21 in about 40% of breast cancers [14, 15]. In addition, the MUC1-C subunit forms auto-inductive interactions with the NF-κB p65 and STAT1/3 transcription factors that confer activation of the *MUC1* promoter and thereby MUC1-C expression in breast cancer cells [16-18]. Studies in breast cancer cells have further supported epigenetic regulation of *MUC1* promoter activation through histone modification and DNA methylation [19]. With loss of apical-basal polarity as found in carcinoma cells, MUC1-C is expressed over the entire cell membrane where it interacts with receptor tyrosine kinases, such as EGFR and HER2, and promotes their activation [20, 21]. The MUC1-C cytoplasmic domain is an intrinsically disordered protein that interacts with multiple effectors, such as PI3K, NF-κB p65 and β-catenin, which have been associated with transformation [12, 22]. In addition and like xCT, MUC1-C has been linked to the regulation of GSH and maintenance of intracellular redox balance [12, 23]. The overexpression of MUC1-C is sufficient to induce anchorage-independent growth and tumorigenicity, supporting its function as an oncoprotein [12]. Other studies have shown that MUC1-C confers self-renewal of breast cancer cells [24]. Thus, targeting MUC1-C with genetic approaches or treatment with inhibitors blocks the capacity of breast cancer cells, including those of the TNBC subtype, to form mammospheres and tumors in mice [24]. These findings have supported the notion that MUC1-C contributes to TNBC cell survival.

The present studies have investigated the potential relationship between MUC1-C and xCT based on the findings that both are of importance for redox balance and self-renewal of TNBC cells [8, 24]. Our results demonstrate that MUC1-C associates with the xCT/CD44v complex in TNBC cells and stabilizes xCT. In turn, we show that targeting xCT suppresses MUC1-C expression by promoting epigenetic modifications of the *MUC1* promoter. Our findings provide further support for a model in which MUC1-C and xCT function in a pathway that regulates ferroptosis and thereby survival of TNBC cells.

## RESULTS

### MUC1-C interacts with xCT

**Figure 1:**
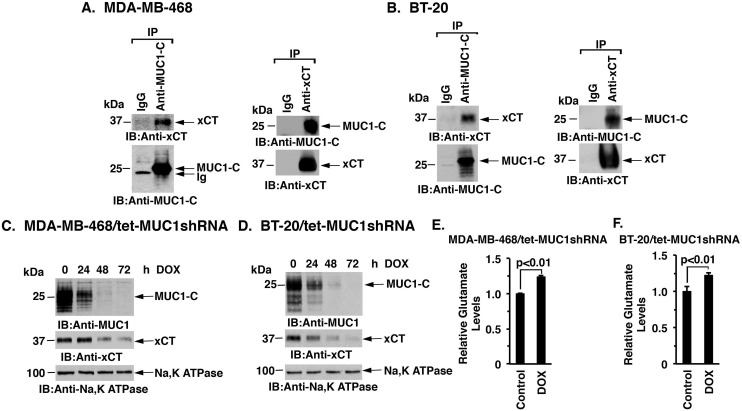
Downregulation of MUC1-C decreases xCT levels **A.** and **B.** Lysates from MDA-MB-468 (A) or BT-20 (B) cells were precipitated with anti-MUC1-C, anti-xCT or a control IgG. The precipitates were immunoblotted with the indicated antibodies. **C.** and **D.** MDA-MB-468/tet-MUC1shRNA (C) and BT-20/tet-MUC1shRNA (D) cells were treated with 200 ng/ml DOX for the indicated times. Membrane fractions were immunoblotted with the indicated antibodies. **E.** and **F.** Intracellular glutamate levels were determined in MDA-MB-468/tet-MUC1shRNA (E) and BT-20/tet-MUC1shRNA (F) cells cultured in the presence of 200 ng/ml DOX for 72 h. The results (mean±SD of 3 determinations) are expressed as relative glutamate levels as compared with that obtained from cells without DOX treatment (assigned a value of 1).

MUC1-C and the xCT antiporter are both aberrantly expressed in TNBC cells [8, 13]; however, there is no known interaction between these two cell membrane proteins. Studies performed with MDA-MB-468 TNBC cells demonstrated that MUC1-C coprecipitates with xCT (Figure 1A, left). The detection of MUC1-C/xCT complexes was confirmed when anti-xCT precipitates were analyzed by immunoblotting with anti-MUC1-C (Figure 1A, right). Similar results obtained with BT-20 TNBC cells provided further support that MUC1-C associates with xCT (Figure 1B, left and right). To assess the functional significance of the MUC1-C/xCT interaction, we generated TNBC cells with tetracycline inducible expression of a MUC1 shRNA (tet-MUC1shRNA) or a control shRNA (tet-CshRNA). Treatment of MDA-MB-468/tet-MUC1shRNA cells with DOX for 48 h was associated with suppression of membrane-associated MUC1-C (Figure 1C) and total cellular MUC1-C (Supplementary Figure S1A). Notably, doxycycline (DOX)-induced MUC1-C suppression in MDA-MB-468/tet-MUC1shRNA cells was also associated with decreases in xCT levels (Figure 1C and Supplementary Figure S1A). By contrast, DOX had no effect on MUC1-C or xCT expression in the control MDA-MB-468/tet-CshRNA cells (Supplementary Figure S1B). Similar results were obtained with DOX-treated BT-20/tet-MUC1shRNA and BT-20/tet-CshRNA cells (Figure 1D and Supplementary Figures S1C and S1D), indicating that silencing MUC1-C downregulates xCT levels. xCT functions in the transmembrane exchange of extracellular cysteine and intracellular glutamate. In concert with the downregulation of xCT, DOX treatment of MDA-MB-468/tet-MUC1shRNA and BT-20/tet-MUC1shRNA cells was associated with significant increases in intracellular glutamate (Figure 1E, left and right). These findings provided support for the notion that targeting MUC1-C promotes the downregulation of xCT expression.

### MUC1-C interacts with the xCT/CD44 complex

**Figure 2:**
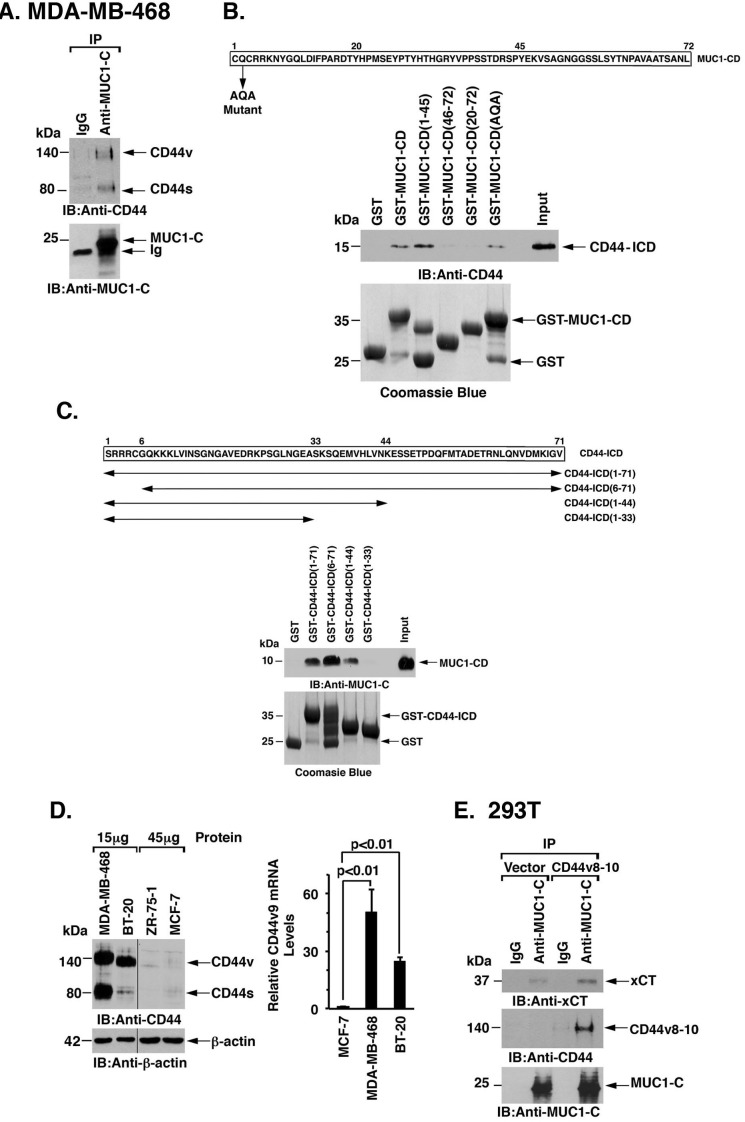
MUC1-C interacts with CD44v **A.** Lysates from MDA-MB-468 cells were precipitated with anti-MUC1-C or a control IgG. The precipitates were immunoblotted with the indicated antibodies. **B.** Amino acid sequence of the MUC1 cytoplasmic domain (MUC1-CD). GST, GST-MUC1-CD or the indicated GST-MUC1-CD fragments were incubated with His-CD44-ICD. The adsorbates were immunoblotted with anti-CD44. Input of the GST proteins was assessed by Coomassie blue staining. **C.** Amino acid sequence of the CD44-ICD. GST, GST-CD44-ICD or the indicated GST-CD44-ICD fragments were incubated with MUC1-CD. The adsorbates were immunoblotted with anti-MUC1-C. Input of the GST proteins was assessed by Coomassie blue staining. **D.** Lysates (15 and 45 μg) from MDA-MB-468, BT-20, ZR-75-1 and MCF-7 cells were immunoblotted with the indicated antibodies (left). CD44v9 mRNA levels in the indicated cells were determined by qRT-PCR (right). The results (mean±SD of 4 determinations) are expressed as relative CD44v9 mRNA levels as compared with that obtained for MCF-7 cells (assigned a value of 1). **E.** Lysates from 293T/MUC1-C cells transfected with an empty vector or one expressing CD44v8-10 were precipitated with anti-MUC1-C or a control IgG. The precipitates were immunoblotted with the indicated antibodies.

xCT forms a complex with the CD44 variant (CD44v) at the cell membrane [7]. Analysis of MDA-MB-468 and BT-20 cells demonstrated that, like xCT, MUC1-C associates with CD44v (Figure 2A and Supplementary Figure S2). To define the nature of the association between MUC1-C and CD44v, we performed binding studies with the MUC1-C cytoplasmic domain (MUC1-CD) and the CD44 intracellular domain (CD44-ICD). The experiments revealed a direct interaction between MUC1-CD and CD44-ICD (Figure 2B). Moreover, using deletion mutants, we found that CD44-ICD binds to MUC1-CD(1-45), but not to MUC1-CD(20-72), indicating that MUC1-CD amino acids 1-19 confer the interaction with CD44-ICD (Figure 2B). In addition, binding studies with CD44-ICD fragments demonstrated that MUC1-CD interacts with CD44-ICD amino acids 34-44 (Figure 2C). The CD44v9 region is of importance for the interaction between CD44v and xCT [7]. We found that the CD44v9 variant is highly expressed in MDA-MB-468 and BT-20 TNBC cells as compared to that in luminal ZR-75-1 and MCF-7 breast cancer cells (Figure 2D, left and right). Accordingly, we asked if CD44v9 contributes to the interaction between MUC1-C and xCT. In support of this notion, transient overexpression of CD44v8-10 in 293T/MUC1-C cells was associated with increases in MUC1-C/xCT complexes as compared to that in 293T/vector cells (Figure 2E). These findings supported a model in which MUC1-C associates with the xCT/CD44v complex, at least in part, through a direct interaction with CD44v.

### MUC1-C promotes xCT stabilization

**Figure 3:**
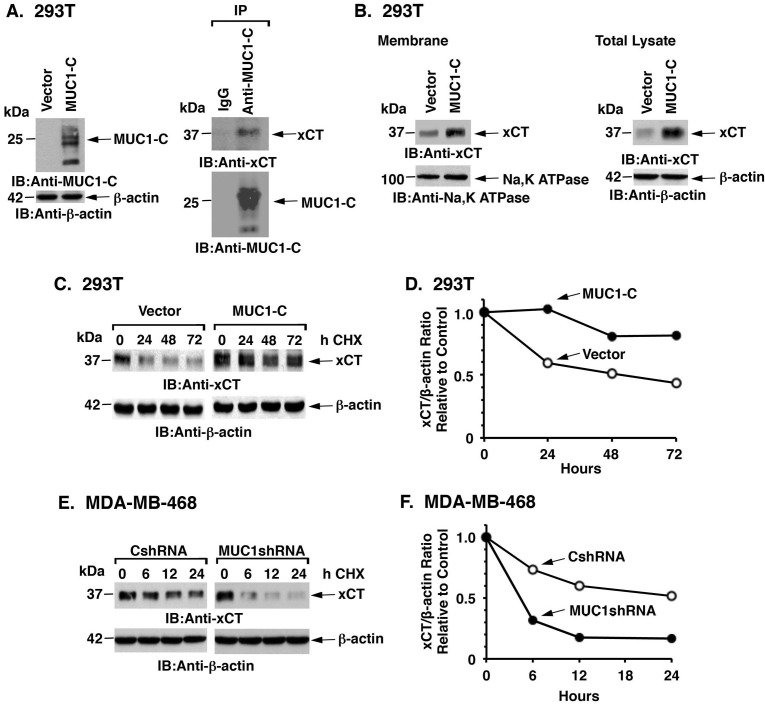
MUC1 increases xCT stability **A.** 293T cells were transfected to stably express MUC1-C or a control vector. Lysates from the transduced cells were immunoblotted with the indicated antibodies (left). Lysates from 293T/MUC1-C cells were precipitated with anti-MUC1-C or a control IgG. The precipitates were immunoblotted with the indicated antibodies (right). **B.** Membrane fractions (left) and total cell lysates (right) from 293T/vector or 293T/MUC1-C cells were immunoblotted with the indicated antibodies. **C.** 293T/vector and 293T/MUC1-C cells were exposed to 50 μg/ml CHX for the indicated times. Total cell lysates were immunoblotted with the indicated antibodies. **D.** Intensities of the xCT signals as compared to those obtained for β-actin (xCT/β-actin ratio) for the CHX-treated 293T/vector and 293T/MUC1-C cells are plotted relative to the control (time 0; assigned a value of 1). **E.** MDA-MB-468/CshRNA and MDA-MB-468/MUC1shRNA cells were exposed to CHX (50 μg/ml) for the indicated times. Total cell lysates were immunoblotted with the indicated antibodies. **F.** Intensities of the xCT signals as compared to those obtained for β-actin (xCT/β-actin ratio) for the CHX-treated MDA-MB-468/CshRNA and MDA-MB-468/MUC1shRNA cells are plotted relative to the control (time 0; assigned a value of 1).

CD44v stabilizes xCT at the cell membrane and thereby regulates redox balance in cancer cells [7]. To determine whether the interaction of MUC1-C with xCT/CD44v affects xCT expression, we first stably overexpressed MUC1-C in MUC1-null 293T cells (Figure 3A, left). As found in TNBC cells, MUC1-C forms complexes with xCT (Figure 3A, right). We also found that MUC1-C expression is associated with increases in xCT levels in the membrane fraction (Figure 3B, left) and in total cell lysates (Figure 3B, right). Analysis of xCT stability in the presence of cyclohexamide (CHX) further demonstrated that MUC1-C expression is associated with an increase in xCT half-life (Figures 3C and 3D). In concert with these results, silencing MUC1-C in MDA-MB-468 (Figures 3E and 3F) and BT-20 (Supplementary Figures S3A and S3B) cells was associated with a decrease in xCT stability. These results thus provided support for involvement of MUC1-C in the stabilization of xCT.

### Targeting xCT decreases MUC1-C expression

**Figure 4:**
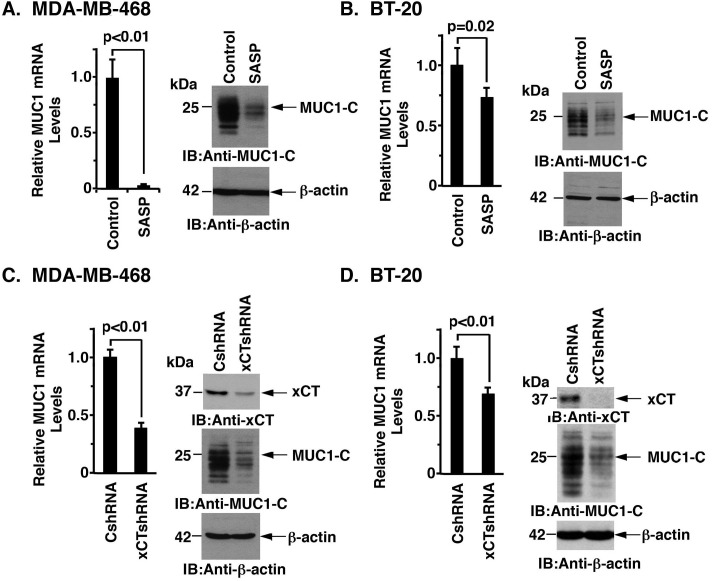
Targeting xCT suppresses MUC1 expression **A.** and **B.** MDA-MB-468 (A) or BT-20 (B) cells were treated with 1.0 μM or 1.5 μM SASP for 72 h, respectively. MUC1 mRNA levels were determined by qRT-PCR (left). The results (mean±SD of 4 determinations) are expressed as relative MUC1 mRNA levels as compared with that obtained for the untreated control cells (assigned a value of 1). Lysates from control and SASP-treated cells were immunoblotted with the indicated antibodies (right). **C.** and **D.** MDA-MB-468 (C) and BT-20 (D) cells were transfected to stably express a control shRNA (CshRNA) or an xCT shRNA (xCTshRNA). MUC1 mRNA levels were determined by qRT-PCR (left). The results (mean±SD of 4 determinations) are expressed as relative MUC1 mRNA levels as compared with that obtained for the CshRNA cells (assigned a value of 1). Lysates were immunoblotted with the indicated antibodies (right).

In experiments assessing the interaction between MUC1-C and xCT, we treated MDA-MB-468 cells with the xCT inhibitor SASP. Surprisingly, SASP treatment was associated with marked suppression of MUC1-C mRNA and protein (Figures 4A, left and right). Significant effects of SASP on MUC1-C expression were also observed in BT-20 cells (Figure 4B, left and right). To provide further evidence that xCT regulates MUC1 expression, we stably silenced xCT and found associated decreases in MUC1 mRNA and protein in MDA-MB-468 (Figure 4C) and BT-20 (Figure 4D) cells. Similar results were obtained with MDA-MB-468 (Supplementary Figure S4A) and BT-20 (Supplementary Figure S4B) cells stably expressing a second xCT shRNA (xCTshRNA-2), indicating that silencing xCT decreases MUC1-C expression.

### Glutamate promotes the downregulation of MUC1-C expression

**Figure 5:**
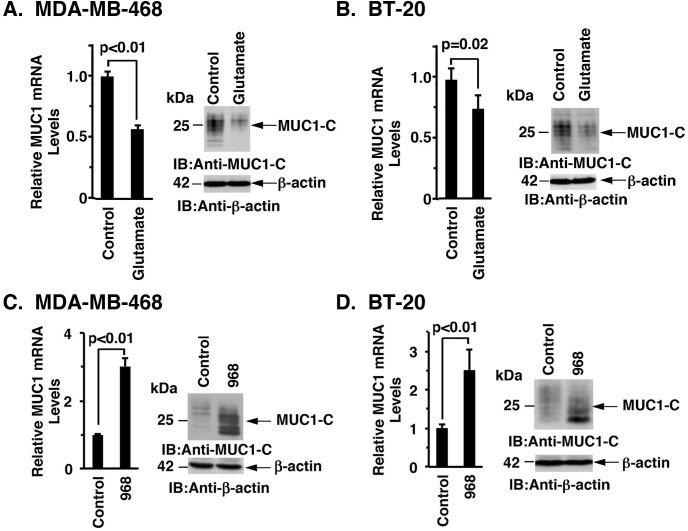
Glutamate suppresses MUC1 expression **A.** and **B.**. MDA-MB-468 (A) or BT-20 (B) cells were treated with 20 mM glutamate for 72 h. MUC1 mRNA levels in the control and glutamate treated cells were determined by qRT-PCR (left). The results (mean±SD of 4 determinations) are expressed as relative MUC1 mRNA levels as compared with that obtained for the untreated control cells (assigned a value of 1). Lysates were immunoblotted with the indicated antibodies (right). **C.** and **D.** MDA-MB-468 (C) or BT-20 (D) cells were treated with 10 μM or 20 μM compound 968 for 72 h, respectively. MUC1 mRNA levels were determined by qRT-PCR (left). The results (mean±SD of 4 determinations) are expressed as relative MUC1 mRNA levels as compared with that obtained for the untreated control cells (assigned a value of 1). Lysates were immunoblotted with the indicated antibodies (right).

As expected, inhibiting xCT with SASP was associated with significant increases in intracellular glutamate levels (Supplementary Figure S5A). Based on these results, we treated MDA-MB-468 cells with glutamate and found downregulation of MUC1-C mRNA and protein (Figure 5A, left and right). Similar results were obtained with BT-20 cells (Figure 5B, left and right). Consistent with these findings, treatment with monosodium glutamate (MSG) similarly resulted in suppression of MUC1-C expression (Supplementary Figures S5B and S5C). The observation that glutamate suppresses MUC1-C expression prompted further studies on the effects of inhibiting the conversion of glutamine to glutamate by glutaminases (GLSs) [25]. Indeed, treatment of MDA-MB-468 cells with the GLS inhibitor Compound 968 was associated with upregulation of MUC1-C mRNA and protein (Figure 5C, left and right). BT-20 cells also responded to Compound 968 with increases in MUC1-C expression (Figure 5D, left and right), supporting a model in which MUC1-C expression is downregulated by targeting xCT and increasing glutamate levels.

### Role of xCT in epigenetic modification of the *MUC1* promoter

**Figure 6:**
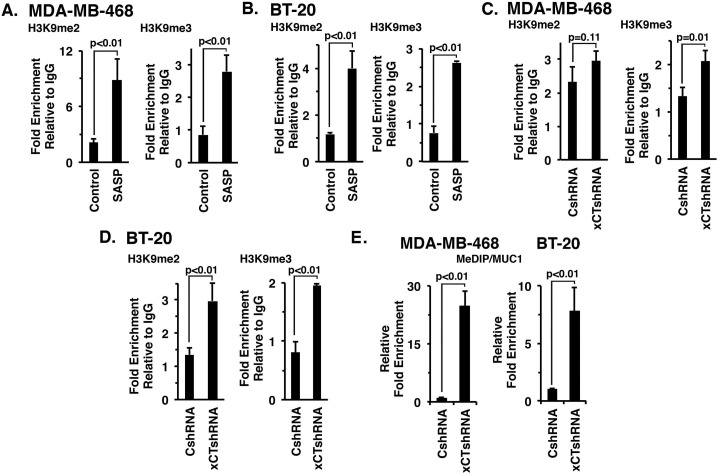
Targeting xCT induces histone and DNA methylation of the *MUC1* promoter **A.** and **B.** MDA-MB-468 (A) or BT-20 (B) cells were treated with 1.0 mM or 1.5 mM SASP for 72 h, respectively. Soluble chromatin from control and SASP treated cells was precipitated with anti-H3K9me2, anti-H3K9me3 or a control IgG. The final DNA samples were amplified by qPCR with pairs of primers for the *MUC1* promoter region or control region from the *GAPDH* promoter. The results (mean±SD of 3 determinations) are expressed as the relative fold enrichment compared with that obtained with the IgG control (untreated cells, assigned a value of 1). **C.** and **D.** Soluble chromatin from MDA-MB-468/CshRNA, MDA-MB-468/xCTshRNA (C), BT-20/CshRNA and BT-20/xCTshRNA (D) cells was precipitated with anti-H3K9me2, anti-H3K9me3 or a control IgG. The final DNA samples were amplified by qPCR with pairs of primers for the *MUC1* promoter region or control *GAPDH* promoter. The results (mean±SD of 3 determinations) are expressed as the relative fold enrichment compared with that obtained with the IgG control (CshRNA cells, assigned a value of 1). **E.** Genomic DNA from the indicated MDA-MB-468 (left) and BT-20 (right) cells was subjected to immunoprecipitation of methylated DNA (MeDIP) and the precipitates were analyzed by qPCR of the *MUC1* gene promoter. The results (mean±SD of 3 determinations) are expressed as relative fold enrichment compared to that obtained from CshRNA expressing cells (assigned a value of 1).

xCT and CD44v can function as a link to epigenetic regulatory mechanisms in cancer cells [26, 27]. Additionally, the epigenetic control of *MUC1* in cancer cells has been associated with H3K9 modification and DNA methylation [19]. Accordingly, we investigated whether targeting xCT with SASP affects H3K9 methylation on the *MUC1* promoter. Indeed, SASP treatment of MDA-MB-468 (Figure 6A, left and right) and BT-20 (Figure 6B, left and right) cells was associated with significant increases in both H3K9me2 and H3K9me3. In concert with these results, we also found that silencing xCT increases H3K9 methylation of the *MUC1* promoter in MDA-MB-468 (Figure 6C, right) and BT-20 (Figure 6D, left and right) cells. In addition, xCT silencing was associated with marked increases in DNA methylation of the *MUC1* promoter (Figures 6E, left and right), consistent with the findings that targeting xCT downregulates MUC1-C expression.

### Targeting the MUC1-C/xCT pathway induces ferroptosis and decreases survival

**Figure 7:**
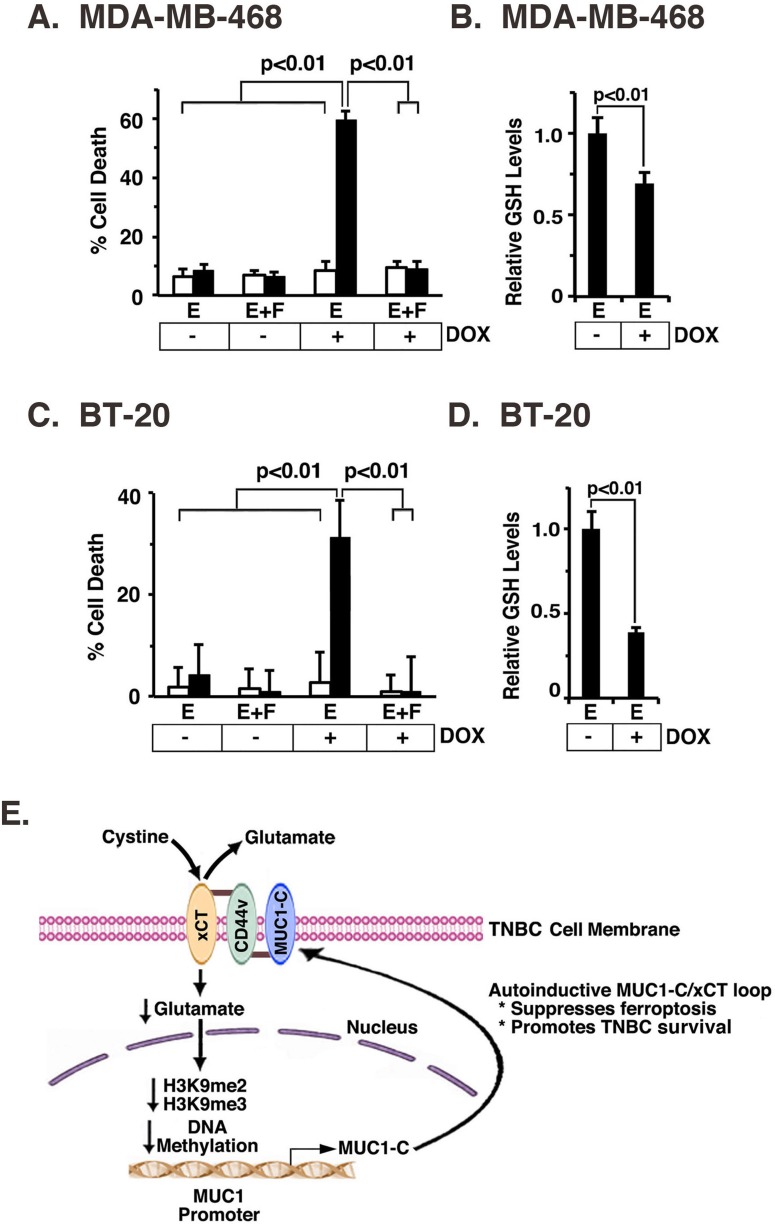
MUC1-C protects against erastin-induced ferroptosis **A.** and **B.** MDA-MB-468/tet-CshRNA (open bars) and MDA-MB-468/tet-MUC1shRNA (solid bars) cells were treated with 200 ng/ml DOX for 7 d. A. Cells were then plated in a 96 well plate, and exposed to 1 μM erastin (labeled E) with or without 2 μM Fer-1 (labeled F) for 24 h. The results (mean±SD of 8 determinations) are expressed as percentage cell death as determined by Alamar blue analysis. B. The indicated cells were analyzed for relative GSH levels (mean±SD of 3 determinations) as compared with that obtained for erastin-treated, DOX- cells (assigned a value of 1)(right). **C.** and **D.** BT-20/tet-CshRNA (open bars) and BT-20/tet-MUC1shRNA (solid bars) cells were treated with 200 ng/ml DOX for 7 d. C. Cells were then plated in a 96 well plate, and exposed to 0.5 μM erastin (labeled E) with or without 2 μM Fer-1 (labeled F) for 12 h. The results (mean±SD of 8 determinations) are expressed as percentage cell death as determined by Alamar blue analysis. D. The indicated cells were analyzed for relative GSH levels (mean±SD of 3 determinations) as compared with that obtained for erastin-treated, DOX- cells (assigned a value of 1)(right). **E.** Schema of the proposed MUC1-C/xCT regulatory loop. MUC1-C forms a complex with xCT and CD44v in the TNBC cell membrane through a direct interaction between the MUC1-C cytoplasmic domain (MUC1-CD) and the CD44v intracellular domain (CD44v-ICD). CD44v interacts with xCT through their extracellular domains [7]. MUC1-C stabilizes xCT; thus targeting MUC1-C decreases xCT in the cell membrane. In addition, targeting xCT with silencing or SASP increases intracellular glutamate and thereby suppresses MUC1 transcription by increasing H3K9 and DNA methylation on the MUC1 promoter. The MUC1-C/xCT loop suppresses ferroptosis and promotes TNBC cell survival.

The small molecule erastin inhibits system X_C_^−^ mediated cystine uptake and induces an iron-dependent form of cell death known as ferroptosis [28]. Based on our findings that MUC1-C stabilizes xCT, we asked if targeting MUC1-C affects erastin-induced ferroptosis. Treatment of MDA-MB-468 cells with erastin for 24 h had no effect on MUC1-C expression (Supplementary Figure S6A) or cell death (Figure 7A). By contrast, exposure to DOX for 7 d and then treatment with erastin was associated a marked induction of cell death in MDA-MB-468/tet-MUC1shRNA, but not MDA-MB-468/tet-CshRNA, cells (Figure 7A). Moreover, we found that erastin-induced death of MDA-MB-468/tet-MUC1shRNA cells with MUC1-C suppression was completely blocked by Fer-1, a lipid ROS scavenger that selectively inhibits ferroptosis [28] (Figure 7A). In concert with the functional importance of MUC1-C, erastin-induced ferroptosis in DOX-treated MDA-MB-468/tet-MUC1shRNA cells was associated with significant decreases in GSH levels (Figure 7B). In addition, we found that BT-20/tet-MUC1shRNA, and not BT-20/tet-CshRNA, cells exposed to DOX were sensitive to erastin, confirming that MUC1-C blocks erastin-induced ferroptosis (Figure 7C and Supplementary Figure S6B) and induces increases in GSH (Figure 7D). These findings supported a model in which the MUC1-C/xCT signaling suppresses ferroptosis and thereby promotes survival of TNBC cells (Figure 7E).

## DISCUSSION

The MUC1-C transmembrane protein is expressed on the apical borders of normal epithelial cells [23]. However, with transformation and loss of polarity, MUC1-C is upregulated and repositioned over the entire cell membrane, where it forms complexes with cell surface receptors normally positioned at the basolateral borders [12]. The present studies demonstrate that MUC1-C associates with the xCT transporter at the cell membrane. Notably, like MUC1-C [12, 13, 23], xCT is highly expressed in diverse malignancies, including TNBC, and is induced by chemotherapy [4-6, 8, 9]. CD44v forms a complex with xCT and in turn stabilizes xCT in gastrointestinal cancer cells [7]. In this respect, our results further indicate that MUC1-C interacts with the xCT/CD44v complex (Figure 7E). The MUC1-C cytoplasmic domain is an intrinsically disordered protein [22], that like other oncogenic molecules in this class [29], has the capacity to interact with diverse effectors and direct the activation of multiple signaling pathways [12]. In concert with this potential, our binding studies demonstrate that the MUC1-C cytoplasmic domain interacts directly with the CD44 intracellular domain (ICD). Expression of CD44 and particularly the variant CD44v isoforms is upregulated in carcinomas and, as an adhesion molecule, plays a role in tumor cell invasion and metastases [7, 30, 31]. CD44v also functions in regulating redox balance in CSCs [26], at least in part by interacting with the xCT extracellular region and thereby stabilizing xCT [7]. In this way, our results support a model in which (i) MUC1-C binds intracellularly to the CD44-ICD, (ii) CD44 interacts extracellularly with xCT, and in turn, (iii) MUC1-C promotes the stabilization of xCT (Figure 7E). Of note, these findings do not exclude the possibility that MUC1-C can also (i) bind to and directly stabilize xCT, or (ii) interact with the system X_C_^−^ 4F2 heavy chain; however, further studies will be needed to address these issues.

MUC1-C is aberrantly overexpressed in breast carcinomas by mechanisms involving in part genetic alterations and dysregulation of transcription [12]. Surprisingly, however, little is known about epigenetic regulation of the *MUC1* gene in cancer cells [19]. In the present studies, we made the unexpected finding that targeting xCT is associated with the downregulation of *MUC1* transcription. Thus, treatment of TNBC cells with the xCT inhibitor SASP decreased MUC1 mRNA and MUC1-C protein. Moreover, silencing xCT with different shRNAs similarly resulted in downregulation of MUC1-C expression, providing initial support for a MUC1-C/xCT positive regulatory loop (Figure 7E). As expected, targeting xCT was associated with increases in glutamate as a result of suppressing the cystine/glutamate transporter function. Accordingly, we found that exposure of TNBC cells to glutamate suppressed MUC1-C expression, invoking the possibility that the glutamate metabolic pathway drives epigenetic downregulation of the *MUC1* promoter. Activation of the *MUC1* promoter has been linked to autoinductive loops involving the (i) NF-κB p65 [32, 33], and (ii) STAT1/3 [18, 34] signaling. However, to our knowledge, there are no reports demonstrating a link between xCT metabolic pathways and epigenetic regulation of *MUC1* promoter. In this context and based on the potential importance of H3K9 modification on the *MUC1* promoter [19], we found that targeting xCT is associated with significant increases in H3K9me2 and H3K9me3 occupancy, supporting a mechanism for suppression of *MUC1* transcription (Figure 7E). In addition, the *MUC1* promoter includes multiple CpG sites that contribute to the regulation of MUC1 expression in response to 5-aza-2′-deoxycytidine [19]. Notably, xCT targeting was also associated with marked induction of DNA methylation on the *MUC1* promoter (Figure 7E). These results thus support the premise that xCT suppresses H3K9 and DNA methylation of the *MUC1* promoter and thereby promotes MUC1-C expression (Figure 7E).

Targeting MUC1-C in breast and other cancer cells is associated with decreases in GSH, disruption of redox balance and ROS-mediated death by mechanisms that have remained unclear [12, 17, 35, 36]. In this respect, stabilization of xCT by CD44v in cancer cells increases intracellular cystine and enhances the capacity for GSH synthesis [7]. Thus, the present findings that targeting MUC1-C decreases xCT stability lend credence to the notion that MUC1-C maintains redox balance by promoting a positive regulatory loop with the xCT pathway (Figure 7E). Implicit in this model is that targeting MUC1-C decreases xCT and thereby induces ROS-mediated death. Based on these premises, we treated TNBC cells with erastin, an inhibitor of cystine uptake by system X_C_^−^ and inducer of ferroptosis, an iron-dependent form of nonapoptotic ROS-mediated cell death [28]. Importantly, erastin was ineffective in inducing ferroptosis in the presence of MUC1-C expression. By contrast, silencing MUC1-C was necessary for erastin-induced ferroptosis, which was substantiated by the suppression of cell death with Fer-1. Our findings therefore indicate that targeting of the MUC1-C/xCT signaling pathway represents a potential therapeutic approach to induce ferroptosis and thereby contribute to inhibition of MUC1-C-mediated TNBC cell self-renewal capacity and tumorigenicity [24] (Figure 7E). These findings of functional interactions between xCT, CD44v and MUC1-C in TNBC cells may also be more broadly applicable to other types of carcinomas.

## MATERIALS AND METHODS

### Cell culture

MDA-MB-468 and MCF-7 breast cancer cells, and 293T embryonic kidney cells were grown in DMEM media supplemented with 10% heat-inactivated FBS (HI-FBS), 100 μg/ml streptomycin, and 100 units/ml penicillin. BT-20 breast cancer cells were grown in EMEM media supplemented with HI-FBS and antibiotics. ZR-75-1 breast cancer cells were grown in RPMI1640 media containing with HI-FBS and antibiotics. MDA-MB-468 and BT-20 cells were infected with a lentiviral vector expressing a MUC1shRNA (Sigma; TRCN0000122938), xCTshRNAs (Sigma; TRCN0000043126, TRCN0000043123) or with a scrambled control shRNA vector (CshRNA; Sigma) as described [37]. 293T cells were transfected to stably express a control pIRES-puro2 or a pIRES-puro2-MUC1-C vector [20]. Cells were treated with doxycycline (DOX), sulfasalazine (SASP), L-glutamate, monosodium glutamate (MSG), Compound 968 (Millipore), erastin (Selleckchem) or ferrostatin 1 (Fer-1) (Tocris Bioscience). Cell viability was determined using the Alamar blue assay as described [38].

### Immunoprecipitation and immunoblotting

Total cell lysates were prepared in lysis buffer as described [36]. Membrane fractions were extracted from whole cell lysates using the Membrane Protein Extraction Kit (BioVision). Soluble proteins were subjected to immunoprecipitation with anti-MUC1-C [39] or anti-xCT (Novus). Precipitates and cell lysates were analyzed by immunoblotting with anti-MUC1-C, anti-xCT anti-β-actin (Sigma), anti-Na,K ATPase and anti-CD44 (Cell Signaling Technology). Immune complexes were detected using horseradish peroxidase-conjugated secondary antibodies and enhanced chemiluminescence (PerkinElmer). Quantification of signal intensity was performed using Image J software.

### Tetracycline-inducible MUC1 silencing

A MUC1shRNA (MISSION shRNA; Sigma, TRCN0000122938) was cloned into the pLKO-tet-puro vector (Addgene, Plasmid #21915). The pLKO-tet-puro vector was co-transfected with the lentivirus packaging plasmids into 293T cells, and the supernatant containing the viral particles was collected at 48 h after transfection. MDA-MB-468 or BT-20 cells were incubated with the supernatant for 12 h in the presence of 8 μg/ml polybrene, followed by replacement with complete cell culture medium and selection in 1-3 μg/ml puromycin.

### Measurement of glutamate levels

Intracellular glutamate levels were measured using the Glutamate Assay Kit (Abcam).

### Transient overexpression of CD44v

CD44v8-10 was generated by RT-PCR using MDA-MB-468 total RNA as a template. The PCR products were digested with EcoRI/XhoI and then cloned into corresponding sites in pcDNA3. 293T cells were transfected with pcDNA3 or pcDNA3-CD44v with SuperFect Transfection Reagent (QIAGEN), and the cells were harvested at 48 h after transfection.

### *In vitro* binding assays

GST, GST-MUC1-CD, GST-MUC1-CD(1-45), GST-MUC1-CD(46-72) GST-MUC1-CD(20-72) and GST-MUC1-CD(AQA) were prepared as described [18, 40]. The CD44 intracellular domain (CD44-ICD) was generated by RT-PCR using SK-CO-1 cell total RNA as a template. The PCR products were digested with EcoRI/XhoI and then cloned into corresponding sites in pGEX-5X-1 (GE Healthcare) to generate GST fusion proteins. Site-directed mutagenesis PCR (Agilent) was performed to generate deletion mutants of CD44-ICD. CD44-ICD PCR products were also digested with EcoRI/XhoI and cloned into corresponding sites in pET-28b to generate His-tagged proteins. Purified GST-MUC1-CD was cleaved with thrombin to remove the GST moiety. For bindings assays, purified proteins were incubated for 2 h at room temperature. Adsorbates to glutathione-conjugated beads were analyzed by immunoblotting.

### Quantitative RT-PCR

cDNA synthesis was performed with 2 μg of total RNA using the SuperScript III First-Strand Synthesis System (Invitrogen). cDNA samples were then amplified using The Power SYBR Green PCR Master Mix (Applied Biosystems) and the 7300 Realtime PCR System (Applied Biosystems)[41]. Primers used for qRT-PCR analysis are included in Supplementary Table S1.

### Chromatin immunoprecipitation (ChIP) assays

Soluble chromatin was prepared from cells as described [42] and precipitated with anti-trimethyl H3K9, anti-dimethyl H3K9 (Abcam) or a control nonimmune IgG. The SYBR green qPCR kit was used for ChIP qPCRs with the ABI Prism 7000 Sequence Detector (Applied Biosystems). Relative fold enrichment was calculated as described [43]. Primers used for qPCR of the *MUC1* promoter and control region are listed in Supplementary Table S2.

### Methylated DNA immunoprecipitation (MeDIP) assays

DNA methylation analysis was performed using the Methylation DNA IP (MeDIP) kit (Active Motif) by immunoprecipitating and enriching for DNA fragments containing 5-mC. Genomic DNA from CshRNA or xCTshRNA expressing cells was digested with Mse-1, heat denatured, and methylated DNA was precipitated with an anti-5-mC antibody (Active Motif). The precipitated DNA was subjected to qPCR analysis using the SYBR green master mix and amplification in an AB7000 sequence detector (Applied Biosystems). Primer sets used to analyze the *MUC1* promoter are listed in Supplementary Table S2.

### Measurement of GSH levels

Intracellular GSH concentrations were determined using the Bioxytech GSH-400 kit (OXIS International) as described.

## SUPPLEMENTARY MATERIAL TABLES AND FIGURES



## Abbreviations

xCT: Xc- light chain
TNBC: triple-negative breast cancer
CD44v: CD44 variant
CD44-ICD: CD44 intracellular domain
CSCs: cancer stem-like cells
MUC1: mucin 1
DOX: doxycycline
SASP: sulfasalazine
MSG: monosodium glutamate
GSH: glutathione
ChIP: chromatin immunoprecipitation
MeDIP: methylated DNA immunoprecipitation
CHX: cyclohexamide
Fer-1: ferrostatin 1
