# Supplementary material for: Functional interactions of the cystine/glutamate antiporter, CD44v and MUC1-C oncoprotein in triple-negative breast cancer cells
Source: Oncotarget. 2016 Feb 22;7(11):11756–69. doi: 10.18632/oncotarget.7598 (PMC4914246; doi:10.18632/oncotarget.7598)
Supplement: Supplementary file 1 [file oncotarget-07-11756-s001.pdf]

Functional interactions of the cystine/glutamate antiporter, CD44V and MUC1-C oncoprotein in triple-negative breast cancer cells

Supplementary Material

Supplementary Table S1. qPCR primer sequences for gene expression.

MUC1 mRNA	Fwd: 5′-TACCGATCGTAGCCCCTATG-3′
	Rev: 5′-CTCACCAGCCCAAACAGG-3′
CD44v9 mRNA	Fwd: 5′-GGCTTGGAAGAAGATAAAGACC-
	Rev: 5′-TGCTTGATGTCAGAGTAGAAGTTG-3′
GAPDH mRNA	Fwd: 5′-CCATGGAGAAGGCTGGGG-3′
	Rev: 5′-CAAAGTTGTCATGGATGACC-3′

Supplementary Table S2. qPCR primer sequences for CHIP and MeDIP.

MUC1 Promoter	Fwd: 5′-GTTTGCGGAGTCCCAGAAG-3′
	Rev: 5′-GGAGAAAACACGAGTAGCTAGGTG-3′
Control (GAPDH)	Fwd: 5′-TACTAGCGGTTTTACGGGCG-3′
	Rev: 5′-TCGAACAGGAGGAGCAGAGAGCGA-3′

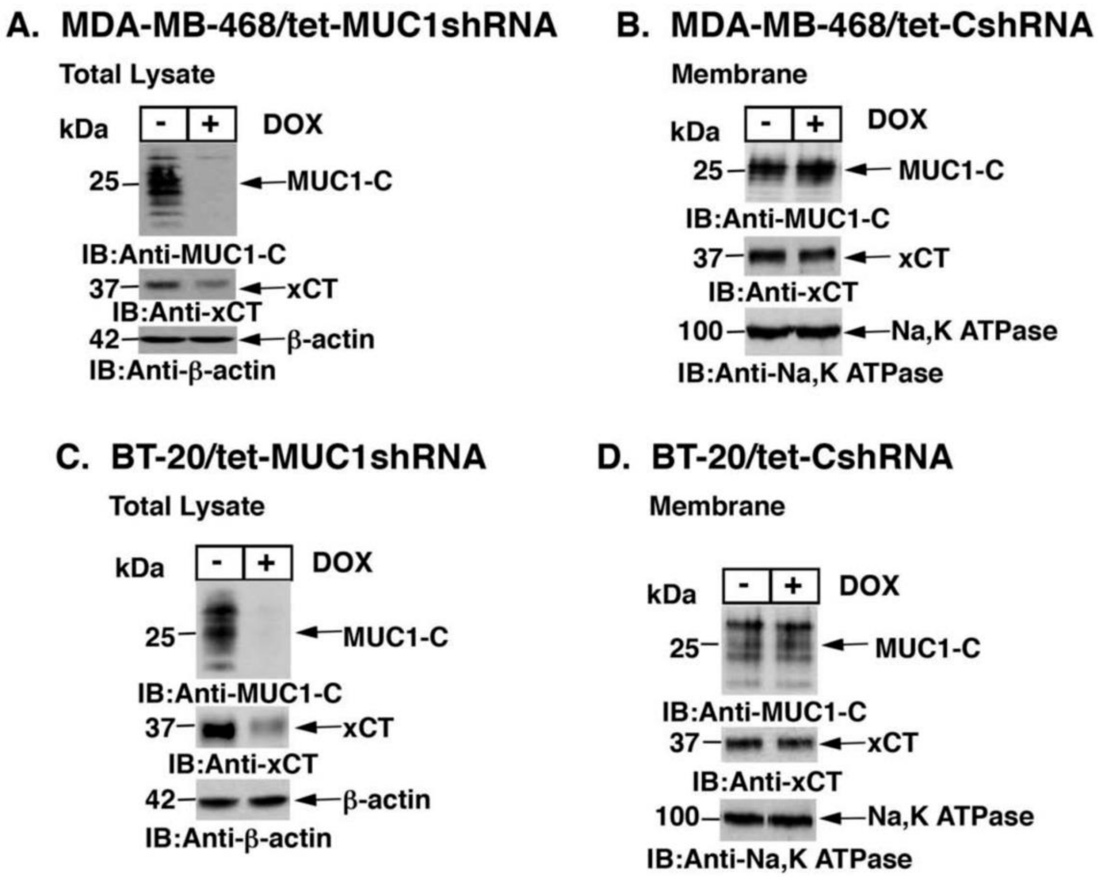

Supplementary Figure S1. A and C. MDA-MB-468/tet-MUC1shRNA (A) and BT-20/tet-MUC1shRNA (C) cells were cultured with or without 200 ng/ml DOX for 48 hours. Total cell lysates were immunoblotted with the indicated antibodies. B and D. MDA-MB-468/tet-CshRNA (B) and BT-20/tet-CshRNA (D) cells were cultured with or without 200 ng/ml DOX for 48 h. Membrane fractions were immunoblotted with the indicated antibodies.

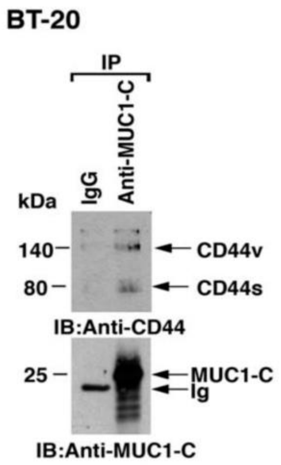

Supplementary Figure S2. Lysates from BT-20 cells were precipitated with anti-MUC1-C or a control IgG. The precipitates were immunoblotted with the indicated antibodies.

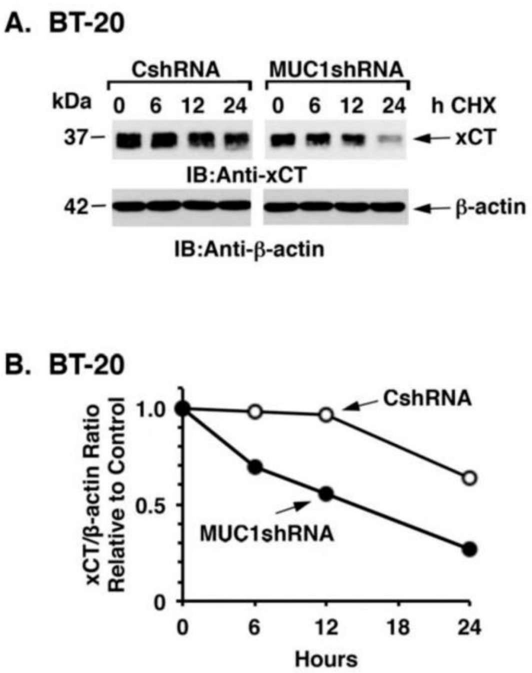

Supplementary Figure S3. A. BT-20/CshRNA and BT-20/MUC1shRNA cells were exposed to CHX (50 μg/ml) for the indicated times. Total cell lysates were immunoblotted with the indicated antibodies. B. Intensities of the xCT signals as compared to those obtained for β-actin (xCT/β-actin ratio) for the CHX-treated BT-20/CshRNA and BT-20/MUC1shRNA cells are plotted relative to the control (time 0; assigned a value of 1).

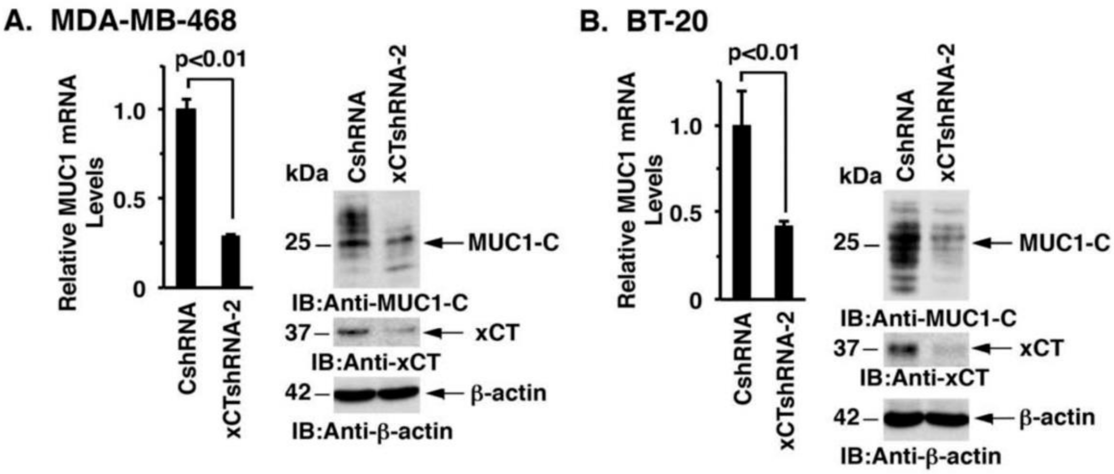

Supplementary Figure S4. A and B. MDA-MB-468 (A) and BT-20 (B) cells were transfected to stably express a control shRNA (CshRNA) or a second xCT shRNA (xCTshRNA-2). MUC1 mRNA levels were determined by qRT—PCR (left). The results (mean±SD of 4 determinations) are expressed as relative MUC1 mRNA levels as compared with that obtained for the CshRNA cells (assigned a value of 1). Lysates were immunoblotted with the indicated antibodies (right).

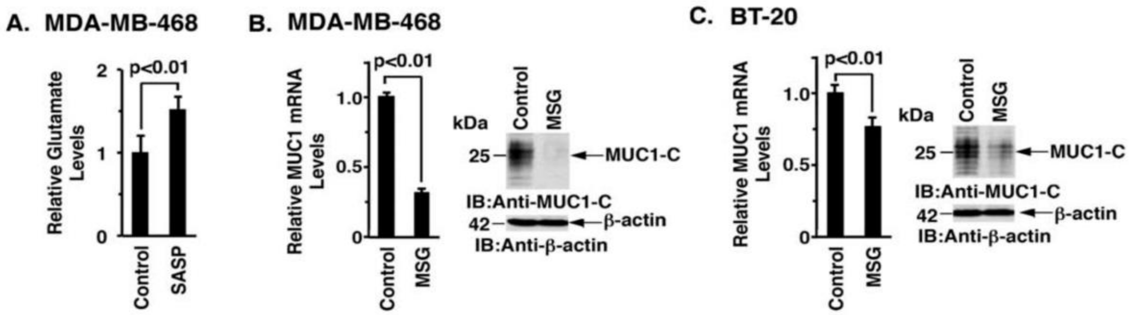

Supplementary Figure S5. A. MDA-MB-468 cells were treated with 1 mM SASP for 72 h and monitored for intracellular glutamate levels. The results (mean±SD of 3 determinations) are expressed as relative to that obtained with the control untreated cells (assigned a value of 1). B and C. MDA-MB-468 (B) or BT-20 (C) cells were treated with 60 Mm monosodium glutamate (MSG) for 72 h. MUC1 mRNA levels in the control and MSG treated cells were determined by qRT-PCR (left). The results (mean±SD of 4 determinations) are expressed as relative MUC1 mRNA levels as compared with that obtained for the untreated control cells (assigned a value of 1). Lysates were immunoblotted with the indicated antibodies (right).

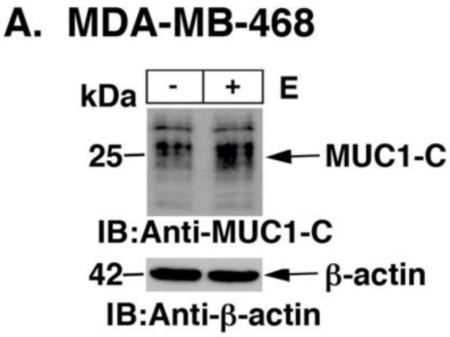

Supplementary Figure S6. A. MDA-MB-468 cells were exposed to 1.0 μM erastin for 24 h. Lysates were immunoblotted with the indicated antibodies. B. BT-20 cells were exposed to 0.5 μM erastin for 12 h. Lysates were immunoblotted with the indicated antibodies.

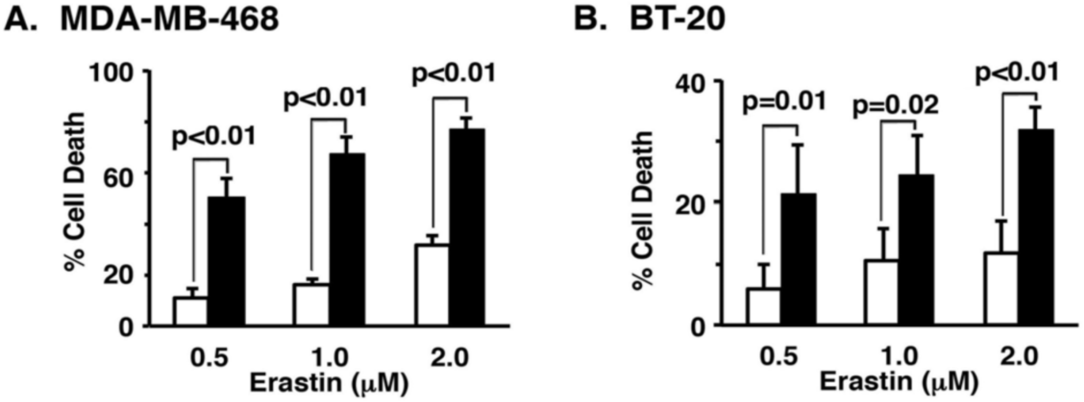

Figure S7. A and B. MDA-MB-468/CshRNA (A; open bars), MDA-MB-468/xCTshRNA (A; solid bars), BT-20/CshRNA (B; open bars) and BT-20/xCTshRNA (B; solid bars) cells were exposed to the indicated concentrations of erastin for 24 h. The results (mean±SD of 4 determinations) are expressed as percentage cell death as determined by Alamar blue analysis.
